# Subspecialized radiological reporting reduces radiology report turnaround time

**DOI:** 10.1186/s13244-020-00917-z

**Published:** 2020-10-30

**Authors:** Andreas Otto Josef Zabel, Sebastian Leschka, Simon Wildermuth, Juerg Hodler, Tobias Johannes Dietrich

**Affiliations:** 1 Division of Radiology and Nuclear Medicine, Kantonsspital St. Gallen, Rorschacherstrasse 95, 9007 St. Gallen, CH Switzerland; 2 Faculty of Medicine, University of Zurich, Pestalozzistrasse 3, CH-8091 Zürich, Switzerland; 3 Department of Diagnostic and Interventional Radiology, University Hospital of Zurich, Rämistrasse 100, 8091 Zürich, CH Switzerland

**Keywords:** Specialization, Change management, Process assessment, health care

## Abstract

**Objectives:**

The objective of this study was to compare the radiology report turnaround time (RTAT) between decentralized/modality-based and centralized/subspecialized radiological reporting at a multi-center radiology enterprise.

**Methods:**

RTAT values for MRI, CT, and conventional radiography were compared between decentralized/modality-based (04 September 2017–22 December 2017) and centralized/subspecialized radiology (03 September 2018–21 December 2018) reporting grouped into three subspecializations (body radiology, musculoskeletal radiology, and neuroradiology) at eleven sites of a multi-center radiology enterprise. For the objective of this investigation, hospitals were defined as major and minor hospitals. The Mann-Whitney *U* test served for statistical analyses.

**Results:**

Change of reporting system from decentralized/modality-based to centralized/subspecialized radiology resulted overall in a significant decrease of the RTAT: from 82 to 77 min for the first signature (*p* < 0.001), and 119 to 107 min and 295 to 238 min for the second signature (*p* < 0.001). Subgroup analyses demonstrate a significant decrease of the RTAT for MRI reports (e.g., second signature RTAT, 1051 to 401 min; *p* < 0.001) and conventional radiographs (e. g., second signature RTAT, 278 to 171 min; *p* < 0.001). The RTAT at major hospitals decreased from 288 to 245 min (second signature; *p* < 0.001) while the corresponding RTAT of minor hospitals decreased more remarkably, from 300 to 198 min (*p* < 0.001). However, the results were heterogenous; in some analyses, the RTAT even increased. The effect size analyses represent small effects.

**Conclusions:**

Change of reporting system from decentralized/modality-based to centralized/subspecialized radiology was associated with a significant decreased RTAT. Specifically, the RTAT for MRI reports and conventional radiographs was significantly reduced. A pronounced RTAT decrease was observed at minor hospitals.

## Key points

- Changing the reporting system can result in a reduced report turnaround time (RTAT).
- RTAT decreased especially for MRI reports and conventional radiographs.
- RTAT of minor hospitals decreased more remarkably compared with major hospitals.

## Introduction

Neuroradiology and pediatric radiology are established subspecializations within the radiology community. In daily practice, further subspecialization is more common in the USA [1] than in most European radiology departments [2]. Subspecialized radiology has been implemented in academic centers [3] but also at larger private and public institutions [1]. Growing radiology networks and teleradiology services are major trends in radiology, which have resulted in significant changes in radiology [4].

**Fig. 1:**
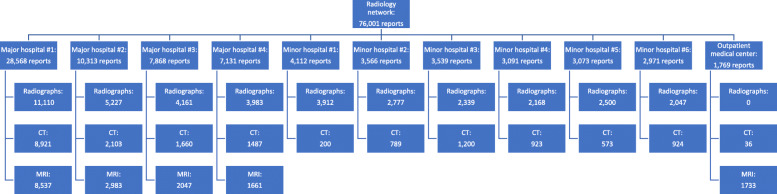
Setup of the investigated radiology network. Numbers of reported examinations summarized for both periods of evaluation for each medical center during standard working hours (4 September–22 December 2017 and 3 September–21 December 2018)

Utilization management of radiological manpower may be more efficient in radiology networks than in smaller units of radiology operating in a number of different hospitals. Furthermore, higher imaging volumes permit the development of subspecialists within one radiology network [5]. Therefore, radiology networks may increase the proportion of subspecialty-focused radiology reporting. Our radiology network consists of eleven radiological sites of various sizes across the entire country (Fig. 1). In the beginning, radiologists were sent to each of the radiological sites and worked in the hospitals using the radiology information system (RIS) for reporting. They were supported by other radiologists using teleradiology. Between 2017 and 2018, a paradigm shift from decentralized/modality-based to centralized/subspecialized reporting was performed. Thereby most of the radiologists were transferred to the main hospital and subspecialization groups were formed while a small number of radiologists remained at the other radiological sites for modality-specific problems and on-site services such as interaction with patients and staff at the hospitals. All imaging studies were divided into different subspecializations and mainly reported by the radiologists within the individual group. We hypothesized that changing the reporting system from general to subspecialized radiological reporting associated with centralization of radiologists reduces the radiology report turnaround time (RTAT).

The aim of this study was to compare the RTAT between decentralized/modality-based and centralized/subspecialized radiological reporting.

## Materials and methods

### Radiology network

The radiology network consists of eleven radiology sites of various sizes, providing radiology services as a part of primary and secondary health care centers for a population of 500,000 individuals (December 2018) [6]. Moreover, the main general hospital provides tertiary health care services for more than 1,000,000 individuals. The setup of the radiology network and the included imaging studies are illustrated in Fig. 1. Major hospitals were defined as hospitals with more than 5000 reports during the examined periods and the availability of at least one MR scanner. The four major hospitals providing inpatient and outpatient medical service are equipped with digital radiography and CT and MR scanners. During the evaluation period of 2018, a second MR scanner was put into service in major hospital #2 (Fig. 1) in addition to the MR scanner already in place since 2017. The six minor hospitals that did not fulfill the two major criteria are providing inpatient and outpatient medical service. They are equipped with digital radiography and a CT scanner but not with a MR scanner. In addition, there is one outpatient medical center in the investigated network equipped with one MR scanner and one PET/CT, which did not fulfill the criteria for neither major nor minor hospitals as it is a dedicated outpatient medical center and not a hospital. The PET/CT is occasionally used for the acquisition of standard CT images only, without positron emission tomography modality*.* The radiology department includes a radiology resident training program. During both investigated periods, as part of the real-life setting, residents got board-certified and new residents were hired. However, there was an unchanged number of residents and board-certified radiologists for both evaluation periods. Residents were trained at the main hospital but could rotate between the several major and minor hospitals. The same RIS (Ana+ 2.3.6879.18755, Cobra Software AG, Arlesheim, Switzerland), PACS (IMPAX EE R20 XVIII v20190821_0813, AGFA HealthCare N. V., Mortsel, Belgium), voice recognition software (Dragon Medical Direct 5.0 (19.1.4741.0), Nuance Communications, Inc, Burlington, MA, USA) [7, 8], and standardized PACS workstations/Hardware and standardized protocols were employed throughout the entire study, both before and after the paradigm shift from decentralized/modality-based to centralized/subspecialized organization.

### Reorganization from modality-/center-based to subspecialized reporting

Until 7 January 2018, the common practice was a decentralized/modality-based workflow with board-certified radiologists or residents responsible for all imaging studies and imaging modalities at each specific site or for a certain imaging modality (radiographs, fluoroscopy, CT, MRI, or ultrasound). Starting in January 2018, the decentralized/modality-based workflow was replaced by centralized radiology with subspecialized teams, supported by teleradiology. The imaging studies of all modalities of the eleven radiology sites were sorted into four subspecializations (body radiology, musculoskeletal radiology, neuroradiology, and pediatric radiology) with each team led by fellowship-trained radiologists. Residents were to be trained in the subspecialized teams and rotated every 6 months. Board-certified radiologists focused on one of the four subspecializations. Reports were acquired by an unchanged number of residents and board-certified radiologists for both periods of evaluation.

The first 9 months (January–September 2018) were considered as a transitional period between both periods of evaluation during which radiologists would adopt the new subspecialization system. Teleradiology-based reporting across the eleven sites was applied to balance radiology report workload among the radiologists at various sites.

### Outcome measures

RTATs were determined by subtracting the time from image acquisition from the first and second electronic signatures of the reports, respectively. Radiology reports can exclusively be viewed by the clinically working doctors after the final signature. Experienced residents were authorized to sign final reports for radiographs but required a final signature from a board-certified radiologist for CT and MRI reports. Board-certified radiologists signed final reports for all imaging modalities but had the opportunity to consult with a senior radiologist for a second signature. RTATs were extracted from the integrated RIS-/PACS database (76,001 reports total). Data analyses of the present study exclusively included radiology examinations performed during core working hours on weekdays (Monday–Friday: 6:54 AM to 16:59 PM) between 4 September 2017 and 22 December 2017 for the decentralized/modality-based system and between 3 September 2018 and 21 December 2018 for the centralized/subspecialized system. Weekends, public holidays, and night shifts were excluded as subspecialized reporting was not feasible due to reduced staffing outside standard working hours. The workflow shift from decentralized/modality-based to centralized/subspecialized radiology reporting did not influence the workflow for nuclear medicine, pediatric radiology, and interventional radiology including CT-guided procedures, fluoroscopy, ultrasound, or mammography. Thus, these procedures and examinations were excluded from the analysis of the present study.

### Statistical analysis

The Kolmogorov-Smirnov test and Q-Q plots indicated that the RTAT values were not normally distributed [9]. Thus, the RTAT values are demonstrated as medians (= 50th percentiles) and 80th percentiles. The 80th percentile RTAT was chosen to determine medium-challenging/time-consuming reports. The Mann-Whitney *U* test served for statistical comparison of RTAT values between decentralized/modality-based radiology system (04 September 2017–22 December 2017) and centralized/subspecialized radiology system (03 September 2018–21 December 2018). The *p* value was adjusted according to the Bonferroni correction [10] because data of the present study underwent multiple testing; thus, a *p* < 0.00121 (= 0.05/41, number of tests *n* = 41) was considered to denote a statistical significant difference between both periods of evaluation. According to Cohen’s guidelines of Pearson’s *r*, effect size (*r*) values smaller than 0.3 were considered to represent a small effect, values between 0.3 and 0.5 were considered to represent a medium effect, and values larger than 0.5 were considered to represent a large effect [11]. Subgroup analyses evaluated imaging modalities such as MRI, CT, and conventional radiographs; subspecializations; and major and minor hospitals. Furthermore, the turnaround time for reports written by residents and board-certified radiologists was compared. Statistical analyses were performed using SPSS (version 25.0.0.1, IBM Corporation, Armonk, NY, USA).

## Results

During the first evaluation period (04 September 2017–22 December 2017), 37,247 imaging examinations (radiography, CT, MRI) were reported by the decentralized/modality-based radiology system during standard working hours. This was compared to 38,754 reports which were acquired during the second evaluation period (03 December 2018–21 December 2018) by the centralized/subspecialized system (3.9% increase). The number of reports with two signatures decreased from 16,632 to 15,967 (4.0% decrease) between the first and second evaluation periods.

### Overall turnaround time

The median RTAT for the first signature was 32 min for both evaluation periods. The 80th percentile RTAT, however, decreased (*p* < 0.001, *r* = 0.02) from 82 to 77 min (Table 1), and the median RTAT for the second signature also decreased (*p* < 0.001, *r* = 0.06), from 119 to 107 min, between the two periods. The corresponding 80th percentile RTAT decreased from 295 to 238 min.

**Table 1:** Overall radiology report turnaround time of all hospitals —04 September 2017–22 December 2017 vs. 03 September 2018–21 December 2018

	Number		Median [min]		80th percentile [min]		***p*** value (***r*** value)
Period	2017	2018	2017	2018	2017	2018	
1st signature	37,247	38,754	32	32	82	77	< .001* (*r* **= 0.02)**
2nd signature	16,632	15,967	119	107	295	238	< .001* (*r* **= 0.06)**

*min* minutes. *Significant *p* value

### Turnaround time for imaging modalities

Subgroup analyses demonstrate a significant difference (*p* < 0.001) of the RTAT between the two evaluation periods for all evaluated imaging modalities (Table 2).

**Table 2:** Comparison of radiology report turnaround time by modalities (all hospitals) —04 September 2017–22 December 2017 vs. 03 September 2018–21 December 2018

	Number		Median [min]		80th percentile [min]		***p*** value (***r*** value)
Period	2017	2018	2017	2018	2017	2018	
**MRI**							
1st signature	8122	8839	45	55	124	112	< .001* (*r* **= 0.09)**
2nd signature	3837	4581	206	163	1051	401	< .001* (*r* **= 0.09)**
**CT**							
1st signature	9058	9758	35	41	78	90	< .001* (*r* **= 0.09)**
2nd signature	4547	4688	87	110	168	214	< .001* (*r* **= 0.15)**
**Conventional radiographs**							
1st signature	20,067	20,157	27	20	71	51	< .001* (*r* **= 0.14)**
2nd signature	8248	6698	117	77	278	171	< .001* (*r* **= 0.21)**

*min* minutes, *MRI* magnetic resonance imaging, *CT* computed tomography. *Significant *p* value

For both first and second signatures, the median RTAT (2017, 27 and 117 min; 2018, 20 and 77 min) and 80th percentile RTAT (2017, 71 and 278 min; 2018, 51 and 171 min) decreased (*p* < 0.001, *r* = 0.14 and 0.21, respectively) for conventional radiographs.

Between the two periods, the median RTAT increased (*p* < 0.001, *r* = 0.09) for the first signature on MRI reports; by contrast, the 80th percentile RTAT for the first signature on MRI reports decreased from 124 to 112 min. Both the median and 80th percentile RTAT for the second signature on MRI reports decreased (*p* < 0.001, *r* = 0.09; median 206 to 163 min; 80th percentile, 1051 to 401 min), while the median RTAT and 80th percentile RTAT for both signatures on CT reports increased (*p* < 0.001, *r* = 0.09 and 0.15, respectively).

### Report turnaround time for subspecialization reporting

Subgroup analyses demonstrate a difference (*p* < 0.001) of the RTAT between the two evaluation periods for all evaluated subspecializations (Table 3).

**Table 3:** Comparison of radiology report turnaround time by subspecializations (all hospitals) —04 September 2017–22 December 2017 vs. 03 September 2018–21 December 2018

	Number		Median [min]		80th percentile [min]		***p*** value (***r*** value)
Period	2017	2018	2017	2018	2017	2018	
**Body radiology**							
1st signature	14,259	14,211	35	33	86	89	< .001* (*r* **= 0.04)**
2nd signature	6969	5428	113	128	252	267	< .001* (*r* **= 0.05)**
**Musculoskeletal radiology**							
1st signature	16,392	17,718	30	26	80	64	< .001* (*r* **= 0.06)**
2nd signature	6419	6898	123	81	300	170	< .001* (*r* **= 0.21)**
**Neuroradiology**							
1st signature	6596	6825	31	44	79	85	< .001* (*r* **= 0.15)**
2nd signature	3244	3641	128	139	409	346	< .001* (*r* **= 0.05)**

*min* minutes. *Significant *p* value

The median and 80th percentile RTAT, for both signatures on body radiology reports, increased (*p* < 0.001, *r* = 0.04 and 0.05, respectively).

For first and second signatures on musculoskeletal radiology reports, the median RTAT (2017, 30 and 123 min; 2018, 26 and 81 min) and 80th percentile RTAT (2017, 80 and 300 min; 2018, 64 and 170 min) decreased (*p* < 0.001, *r* = 0.06 and 0.21, respectively).

Both the median and 80th percentile RTAT for the first signature on neuroradiology reports increased (*p* < 0.001, *r* = 0.15). The median RTAT increased for the second signature on neuroradiology reports; by contrast, the 80th percentile RTAT for the second signature decreased from 409 to 346 min (*p* < 0.001, *r* = 0.05).

### Comparison of major versus minor hospitals

The outpatient medical center did not fulfill the criteria for neither major nor minor hospitals. Thus, the imaging examinations were excluded from the comparison of major versus minor hospitals.

### Overall turnaround time of major versus minor hospitals

Table 4 shows both the median and 80th percentile RTAT for the first signature at the major hospitals increased (*p* < 0.001, *r* = 0.02). By contrast, both the median and 80th percentile RTAT for the first signature at the minor hospitals decreased (*p* < 0.001, *r* = 0.12; median 32 to 24 min, 80th percentile 92 to 62 min). At both major and minor hospitals, for the second signature, both the median and 80th percentile RTAT decreased (*p* < 0.001, *r* = 0.03 and 0.15, respectively). The 80th percentile RTAT decreased at major hospitals from 288 to 245 min and decreased at minor hospitals from 300 to 198 min.

**Table 4:** Comparison of major vs. minor hospitals’ radiology report turnaround time by modalities —04 September 2017–22 December 2017 vs. 03 September 2018–21 December 2018

Major hospitals								Minor hospitals						
	***n***		Median [min]		80th percentile [min]		***p*** value (***r*** value)	***n***		Median [min]		80th percentile [min]		***p*** value (***r*** value)
**Period**	**2017**	**2018**	**2017**	**2018**	**2017**	**2018**		**2017**	**2018**	**2017**	**2018**	**2017**	**2018**	
**All modalities**														
1st signature	26,524	27,356	32	34	78	81	< .001* (*r* **= 0.02)**	9906	10,446	32	24	92	62	< .001* (*r* **= 0.12)**
2nd signature	13,195	11,868	117	110	288	245	< .001* (*r* **= 0.03)**	3270	3663	125	91	300	198	< .001* (*r* **= 0.15)**
**CT**														
1st signature	6897	7274	33	42	71	92	< .001* (*r* **= 0.14)**	2150	2459	40	38	108	83	.01* (*r* **= 0.04)**
2nd signature	3799	3677	86	109	162	212	< .001* (*r* **= 0.16)**	746	997	93	111	204	224	< .001* (*r* **= 0.09)**
**Conventional radiographs**														
1st signature	12,311	12,170	26	19	63	49	< .001* (*r* **= 0.13)**	7756	7987	30	20	88	54	< .001* (*r* **= 0.16)**
2nd signature	5724	4032	110	73	248	156	< .001* (*r* **= 0.21)**	2524	2666	137	83	331	189	< .001* (*r* **= 0.23)**

*min* minutes, *CT* computed tomography. *Significant *p* value

### Turnaround time for imaging modalities of major versus minor hospitals

Subgroup analyses show a difference for the RTAT of the evaluated imaging modalities at major and minor hospitals between the first and second evaluation periods (Table 4).

Most interestingly, the 80th percentile RTAT of the second signature on conventional radiographs decreased at major hospitals from 248 to 156 min, while it decreased from 331 to 189 min at minor hospitals (*p* < 0.001, *r* = 0.21 and 0.23, respectively).

### Turnaround time for subspecialization reporting of major versus minor hospitals

Subgroup analyses demonstrate a difference between the RTAT of the evaluated subspecializations at major and minor hospitals between the first and second evaluation periods (Table 5).

**Table 5:** Comparison of major vs. minor hospitals’ radiology report turnaround time by subspecializations —04 September 2017–22 December 2017 vs. 03 September 2018–21 December 2018

	Major hospitals							Minor hospitals						
	***n***		Median [min]		80th percentile [min]		***p*** value (***r*** value)	***n***		Median [min]		80th percentile [min]		***p*** value (***r*** value)
**Period**	**2017**	**2018**	**2017**	**2018**	**2017**	**2018**		**2017**	**2018**	**2017**	**2018**	**2017**	**2018**	
**Body radiology**														
1st signature	10,722	10,591	35	36	83	94	.46 **(*r*** **= 0.01)**	3368	3342	34	25	93	66	< .001* (*r* **= 0.15)**
2nd signature	5821	4295	115	131	256	277	< .001* (*r* **= 0.06)**	1114	1001	105	107	231	217	.61 **(*r*** **= 0.01)**
**Musculoskeletal radiology**														
1st signature	10,042	10,915	29	27	74	66	< .001* (*r* **= 0.02)**	6096	6553	31	23	94	59	< .001* (*r* **= 0.12)**
2nd signature	4374	4343	114	79	269	157	< .001* (*r* **= 0.20)**	1997	2464	145	84	344	192	< .001* (r = 0.24)
**Neuroradiology**														
1st signature	5760	5850	30	44	77	86	< .001* (*r* **= 0.16)**	442	551	26	31	78	65,6	.22 **(*r*** **= 0.04)**
2nd signature	3000	3230	131	141	423	346	< .001* (*r* **= 0.05)**	159	198	73	106	147	185	.004 **(*r*** **= 0.15)**

*min* minutes. *Significant *p* value

Most interestingly, the median and 80th percentile RTAT for musculoskeletal radiology reports decreased for both signatures at major (*p* < 0.001, *r* = 0.02 and 0.20, respectively) and minor (*p* < 0.001, *r* = 0.12 and 0.24, respectively) hospitals (80th percentile: major hospitals from 269 to 157 min, minor hospitals from 344 to 192 min).

### Turnaround time for residents versus board-certified radiologists

Interestingly, subgroup analyses demonstrate a difference between the RTAT of reports written by board-certified radiologists between the first and second evaluation periods (Table 6). For the first signature, the median RTAT decreased from 29 to 27 min and the 80th percentile RTAT decreased from 80 to 69 min (*p* < 0.001, *r* = 0.05) between the two periods. For the second signature, the median RTAT decreased from 119 to 107 min, while the corresponding 80th percentile RTAT decreased from 296 to 238 min (*p* < 0.001, *r* = 0.06).

**Table 6:** Comparison of radiology report turnaround time residents versus board-certified radiologists—04 September 2017–22 December 2017 vs. 03 September 2018–21 December 2018

	Number		Median [min]		80th percentile [min]		***p*** value (***r*** value)
Period	2017	2018	2017	2018	2017	2018	
**Residents**							
1st signature	17,037	18,109	36	37	85	86	.013 (*r* = 0.01)
**Board-certified radiologists**							
1st signature	20,132	20,584	29	27	80	69	< .001* (*r* = 0.05)
2nd signature	16,551	15,862	119	107	296	238	< .001* (*r* = 0.06)

*min* minutes. *Significant *p* value

By contrast, there was no significant difference between the median and 80th percentile RTAT of reports written by residents (*p* < 0.013, *r* = 0.01) between the two evaluation periods.

## Discussion

The most important finding of this study is that changing the reporting system from decentralized/modality-based radiology to centralized/subspecialized radiology resulted in a significant decrease of the RTAT. Overall, the RTAT decreased from 82 to 77 min (80th percentile) for the first signature (*p* < 0.001), while it decreased from 119 to 107 min (median) and from 295 to 238 min (80th percentile) for the second signature (*p* < 0.001). Subgroup analyses demonstrated a significant decrease of the RTAT for MRI reports (e.g., second signature, 80th percentile RTAT, 1051 to 401 min; *p* < 0.001) and conventional radiographs (e. g., second signature, 80th percentile RTAT, 278 to 171 min; *p* < 0.001).

It has to be noted that the effect size analyses correspond to small effects. Notably, minor hospitals benefited most from the change to centralized/subspecialized radiology, as the RTAT decreased overall from 300 to 198 min (second signature, 80th percentile; *p* < 0.001), while the corresponding RTAT of major hospitals decreased, less remarkably, from 288 to 245 min (*p* < 0.001).

Similarly, to other recently published surveys, the present study demonstrated an annual increase of 3.9% in the number of radiology reports and imaging examinations during the evaluation period in 2018 compared to 2017 (Table 1) [3, 12, 13]. Interestingly, there was an increase from 37,247 to 38,754 reports but a decrease in reports with a second signature from 16,632 to 15,967 reports (Table 1). During the period of decentralized/modality-based radiology, radiologists occasionally forwarded difficult reports to specialists for a second opinion and a second signature, respectively. One may speculate that centralized/subspecialized radiology increases the experience and confidence of radiologists in their dedicated tasks. Thus, centralized/subspecialized radiology may decrease the necessity for requesting a second opinion for challenging radiology reports. The increasing number of radiology reports of 3.9% between the evaluation periods 2017 and 2018 can be considered as the normal annual growth rate of our radiology department.

Thus, as hypothesized, it appears that the RTAT decreased in spite of the increased workload. One may assume that the experience gained by residents and board-certified radiologists may also have improved the RTAT values. Nonetheless, new residents were hired and residents got board-certified as part of the real-life setting. Nonetheless, there was an unchanged number of residents and board-certified radiologists for both evaluation periods. Interestingly, after changing the reporting system from decentralized/modality-based radiology to centralized/subspecialized radiology, the RTAT for reports written by board-certified radiologists decreased significantly, while there was no significant difference of the RTAT for reports written by residents (Table 6). As board-certified radiologists unlike residents focus on one of the four subspecializations, it may also indicate an improvement between the two investigated systems of radiological reporting.

Recent literature revealed conflicting data regarding the RTAT: Change of reporting system from decentralized/modality-based radiology to centralized/subspecialized radiology demonstrated both a decrease of the RTAT, as shown by the results of Stern et al. [14], and, conversely, an increase of the RTAT, as shown by the results of Meyl et al. [3]. In the present study, the change to a centralized/subspecialized system of radiological reporting leads overall to a significant decrease of the RTAT (Tables 1 and 4). The RTAT for MRI reports and conventional radiographs decreased most significantly (Tables 2 and 4), which other studies confirm [14]. In some important and critical areas, significant increases of the RTAT were noted, e.g., for the median of the first signature of MR reports (Table 2), for both signatures of body radiology (Table 3), for the first signature of neuroradiology (Table 3), and for the first signature of major hospitals (Table 4). This may be explained by redistributive effects. Not all areas of this heterogeneous survey benefited from the system change to centralized/subspecialized radiological reporting.

Before the change management from decentralized/modality-based radiology to centralized/subspecialized radiology, the radiology network showed a very low RTAT (Table 1) compared with peer-valued studies [14], which could be improved even further after the change (Table 1). Interestingly, there was an increase for both signatures of the RTAT for CT reports (Table 2) which may be explained because of its very complex or time-consuming cases, by a work redistribution in favor of MRI reports and by general redistribution effects from the management. Time-critical CT reports (e.g., stroke, trauma) were given higher priority than MR reports or reports for conventional radiographs in both evaluated systems of radiological reporting. In addition, a senior staff radiologist was in duty to identify time-sensitive examinations and, if necessary, to distribute them along the radiologists to provide fast reports for emergency and urgent cases. However, this study did not differentiate between emergency and routine CT examinations.

In contrast to the results of Meyl et al. [3], the current study showed particularly that the RTAT for medium-challenging/time-consuming reports, represented by the 80th percentile RTAT, decreased significantly; this is especially true of the RTAT for the subspecialization of musculoskeletal radiology. In the other subspecializations, however, the trend tended towards increased RTATs (Tables 3 and 5), which was also observed by other recent studies [3].

Furthermore, as suspected, there was a connection between the size of hospitals and change of reporting system. Notably, minor hospitals noted a general improvement of the RTAT overall, as well as a significant improvement of the RTAT for conventional radiographs and CT scans (Table 4). Furthermore, minor hospitals benefited from a decreased RTAT for the body and musculoskeletal subspecializations (Table 5). The decreased RTAT may demonstrate a better distribution of workload among all radiologists through the use of teleradiology, in spite of the more centralized and subspecialized multi-center radiology network [15–17].

The findings of this study differ from the other cited surveys, as it provides RTATs not only for single academic or public hospitals but for a multi-center radiology network consisting of eleven radiology sites—which represent public hospitals of various sizes (Fig. 1)—as well as a fully integrated diagnostic neuroradiology department, which is an unusual setup compared to most larger European radiology departments [2].

The limitations of this statistical evaluation were many confounding variables including the experience of radiologists, case complexity, and case volume. Furthermore, as part of the real-life setting, the number of studies included by modality was not equitable as the number of conventional radiographs exceeds the number of CT and MRI reports. Although RTAT may not be considered as the best measure to determine efficiency of a radiology department, it is a frequently used scale for the evaluation of the workflow of many radiology institutions [18–21] as well as the 80th percentile RTAT [14, 22, 23]. Outlier (= very complex or time-consuming cases) such as cardiovascular CT or MR examinations were difficult to define. However, these examinations occurred in both systems of radiological reporting and are part of everyday clinical practice in large institutions. Regarding distortion caused by outliers, the median serves as a robust measurement [24]. According to Cohen’s guidelines, most results of the study show a small effect size. However, there are indications to consider these normative guidelines and to use correlations of 0.10, 0.20, and 0.30 to represent relatively small, typical, and relatively large effects [25].

A study comparing the quality of radiology reports between decentralized/modality-based radiology and centralized/subspecialized radiology was conducted and will be published as a separate study.

In conclusion, changing the reporting system from decentralized/modality-based radiology to a centralized/subspecialized radiology was associated with a significant decrease of the RTAT overall and a significant decrease of the RTAT for MRI reports and conventional radiographs. A significant decrease of the RTAT at minor hospitals, organized in a multi-center radiology network, was also noted—a significant improvement between the two evaluation periods. The effect size corresponds to small effects.

## Data Availability

The full SPSS datasets can be uploaded if it is desired.

## Abbreviations

CT: Computed tomography
MRI: Magnetic resonance imaging
PACS: Picture archiving and communication system
PET/CT: Positron emission tomography CT
RIS: Radiological information system
RTAT: Radiology report turnaround time
